# Supplementary material for: TRPV4 Stimulation Level Regulates Ca2+-Dependent Control of Human Corneal Endothelial Cell Viability and Survival
Source: Membranes (Basel). 2022 Feb 28;12(3):281. doi: 10.3390/membranes12030281 (PMC8952823; doi:10.3390/membranes12030281)
Supplement: Supplementary file 1 [file membranes-12-00281-s001.zip › membranes-1576125-Suipplementary.pdf]

membranes

Supplemental material to

TRPV4 Stimulation Level Regulates Ca^{2+}-Dependent Control of Human Corneal Endothelial Cell Viability and Survival

Jennifer Donau [1,2], Huan Luo [3], Iiris Virta [3], Annett Skupin [1,2], Margarita Pushina [1], Jana Loeffler [1], Frauke V. Haertel [4,5], Anupam Das [5], Thomas Kurth [6], Michael Gerlach [7], Dirk Lindemann [2], Peter S. Reinach [8], Stefan Mergler [3,*] and Monika Valtink [1,9,*]

[1] Institute of Anatomy, Faculty of Medicine, TU Dresden, 01307 Dresden, Germany; jennifer.donau@tu-dresden.de (J.D.); annett.skupin@tu-dresden.de (A.S.); margo171190@gmail.com (M.P.); janaloeffler@web.de (J.L.)
[2] Institute of Medical Microbiology and Virology, Faculty of Medicine, TU Dresden, 01307 Dresden, Germany; dirk.lindemann@tu-dresden.de
[3] Klinik für Augenheilkunde, Charité—Universitätsmedizin Berlin, Corporate Member of Freie Universität Berlin, Humboldt-Universität zu Berlin, and Berlin Institute of Health, 13353 Berlin, Germany; huan.luo@charite.de (H.L.); iiris.virta@hotmail.com (I.V.)
[4] Institute of Physiology, Faculty of Medicine, University Giessen, 35392 Giessen, Germany; frauke.haertel@physiologie.med.uni-giessen.de
[5] Institute of Physiology, Faculty of Medicine, TU Dresden, 01307 Dresden, Germany; anupam.das@tu-dresden.de
[6] Center for Molecular and Cellular Bioengineering (CMCB), Technology Platform, TU Dresden, 01307 Dresden, Germany; thomas.kurth@tu-dresden.de
[7] Core Facility Cellular Imaging, Faculty of Medicine, TU Dresden, 01307 Dresden, Germany; michael.gerlach2@tu-dresden.de
[8] School of Ophthalmology and Optometry, Wenzhou Medical University, Wenzhou 325027, China; preinach25@gmail.com
[9] Equality and Diversity Unit, Faculty of Medicine, TU Dresden, 01307 Dresden, Germany
* Correspondence: stefan.mergler@charite.de (S.M.); monika.valtink@tu-dresden.de (M.V.)

Citation: Donau, J.; Luo, H.; Virta, I.; Skupin, A.; Pushina, M.; Loeffler, J.; Haertel, F.V.; Das, A.; Kurth, T.; Gerlach, M.; et al. TRPV4 Stimulation Level Regulates Ca^{2+}-Dependent Control of Human Corneal Endothelial Cell Viability and Survival. *Membranes* **2022**, *12*, 281. https://doi.org/10.3390/membranes12030281

Academic Editors: Tadashi Kimura and Anna Weronika Sobańska

Received: 13 January 2022
Accepted: 24 February 2022
Published: 28 February 2022

Publisher's Note: MDPI stays neutral with regard to jurisdictional claims in published maps and institutional affiliations.

Copyright: © 2022 by the authors. Licensee MDPI, Basel, Switzerland. This article is an open access article distributed under the terms and conditions of the Creative Commons Attribution (CC BY) license (https://creativecommons.org/licenses/by/4.0/).

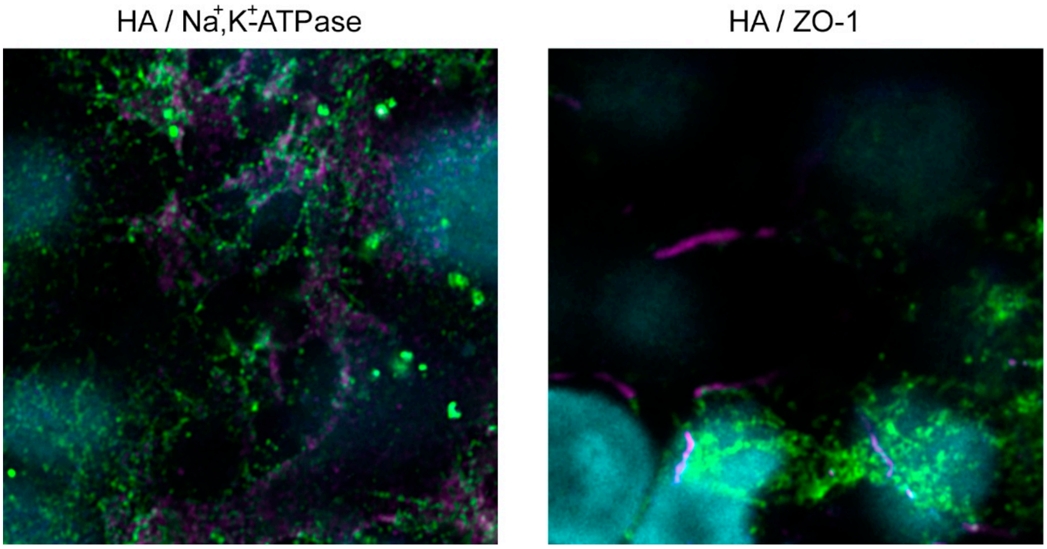

(a) **(b)**

Figure S1: Overviews of the z-stacks presented in the supplemental videos M1 and M2, showing double staining of the HA-tag with either Na^+,K^+-ATPase (**a**) or ZO-1 (**b**). High resolution fluorescent images were acquired at the Core Facility Cellular Imaging with a Zeiss LSM 880 equipped with an AiryScan detector. Imaging was performed in "Superresolution" mode of AiryScan detector at zoom factor 7 and master gain of 800, utilizing an LD LCI Plan-Apochromat 40x/1.2 Imm Korr DIC M27 objective. HA-tag: green fluorescence, Na^+,K^+-ATPase or ZO-1: red fluorescence, nuclei: blue fluorescence.

Video M1: Z-stack videoclip made from S1 (left), showing distribution of HA-TRPV4 and Na^{+},K^{+}-ATPase. 5 frames per second. Scale bar 10 µm

Video M2: Z-stack videoclip made from S1 (right), showing distribution of HA-TRPV4 and ZO-1. 5 frames per second. Scale bar 10 µm

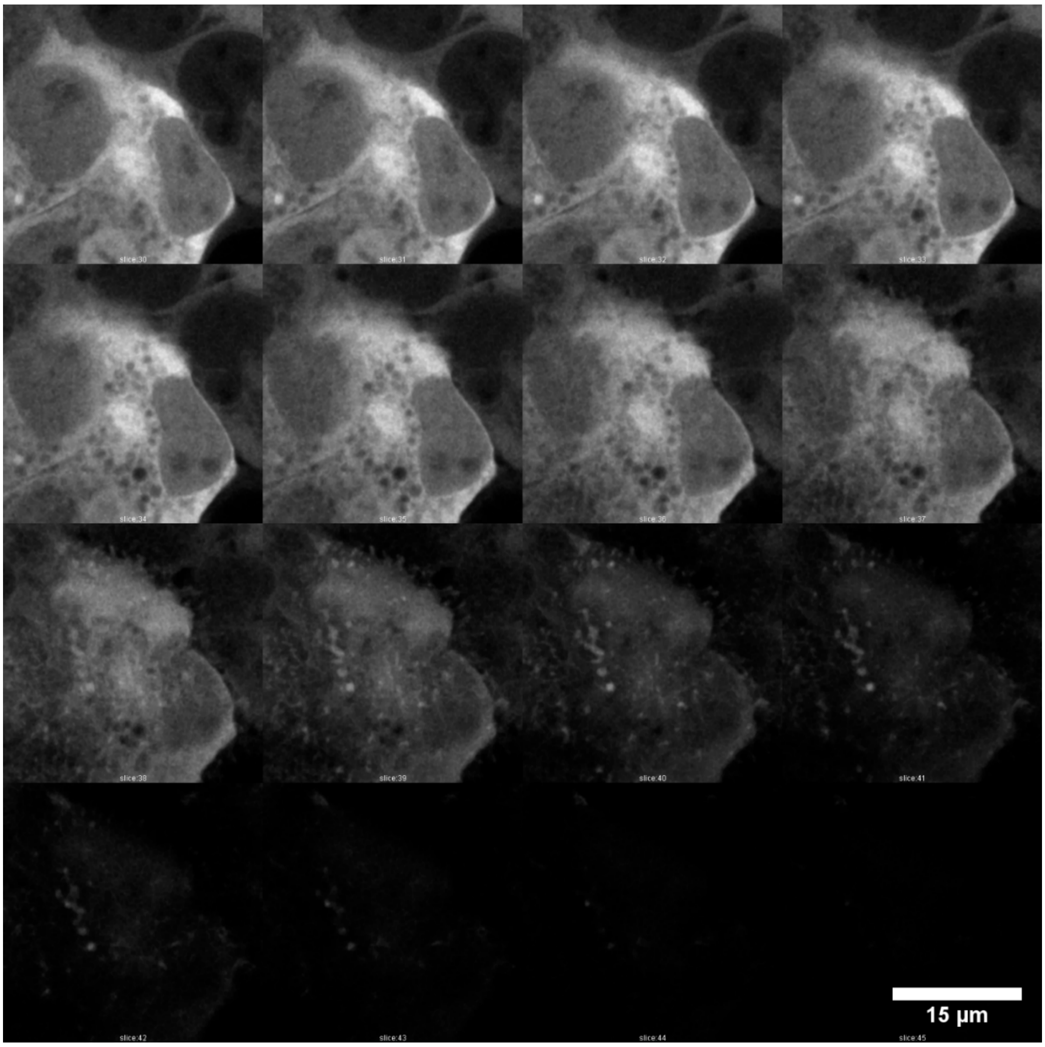

Figure S2: Z-stack of TRPV4 overexpressing (12V4) cells. Fluorescence of the reporter protein dsRedEx2 illustrates villus-like extensions from the apical cell surface. Living, unfixed cells grown on chamber slides were imaged with an inverted Zeiss LSM 880 microscope equipped with an Airyscan detector run by ZEN software (Carl Zeiss Microscopy GmbH, Jena, Germany). Voxel size corresponds to 73 nm × 73 nm × 277 nm, Objective: LD LCI Plan-Apochromat 40x/1.2 Imm Korr DIC M27

Video M3: Z-stack videoclip made from S1, 2 frames per second.

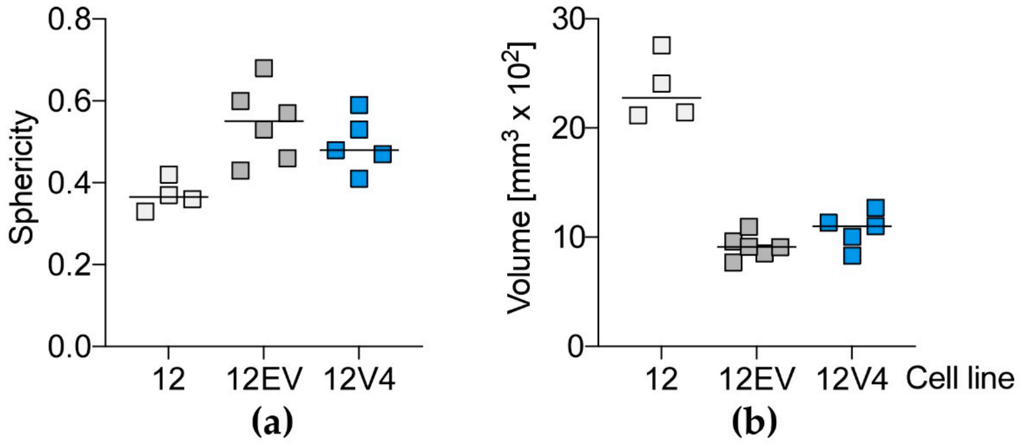

Figure S3: Cell volume and sphericity plots. 3D cell volume analysis was performed semi-automatically in Imaris 9.5.1 (Bitplane AG). 3D stacks of cell monolayers were inspected in a single plane for segmentable cells. Contours of these cells were then manually outlined for each z-plane, following each cell throughout the complete stack. After completion, 3D volume was rendered by Imaris "Surface" algorithm, volume and sphericity was calculated by Imaris. Since quantification of cell volume is difficult in confluent state without clear marker for cell boundaries, the quantification method employed can potentially be biased towards reliably segmentable (i.e. not fully connected or confluent) cells. This, however, applies to all three cell lines and analysis was performed by the same operator on one day to reduce bias.
